# Verbal learning impairment in euthymic bipolar disorder: BDI v BDII

**DOI:** 10.1016/j.jad.2015.04.021

**Published:** 2015-08-15

**Authors:** Corin Bourne, Amy Bilderbeck, Rebecca Drennan, Lauren Atkinson, Jonathan Price, John R. Geddes, Guy M. Goodwin

**Affiliations:** a Department of Psychiatry, University of Oxford, Oxford, UK; b Department of Psychology, Counselling & Psychotherapy, Newman University, Birmingham, UK

**Keywords:** Bipolar disorder subtypes, Cognitive impairment, Verbal learning and memory

## Abstract

**Objectives:**

Cognitive impairment is known to occur in bipolar disorder (BD), even in euthymic patients, with largest effect sizes often seen in Verbal Learning and Memory Tasks (VLT). However, comparisons between BD Type-I and Type-II have produced inconsistent results partly due to low sample sizes.

**Methods:**

This study compared the performance of 183 BDI with 96 BDII out-patients on an adapted version of the Rey Verbal Learning Task. Gender, age, years of education, mood scores and age at onset were all used as covariates. Current medication and a variety of illness variables were also investigated for potential effects on VLT performance.

**Results:**

BDI patients were significantly impaired relative to BDII patients on all five VLT outcome measures after controlling for the other variables [Effect Sizes=.13–.17]. The impairments seem to be unrelated to drug treatment and largely unrelated to illness variables, although age of onset affected performance on three outcome measures and number of episodes of mood elevation affected performance on one.

**Limitations:**

This study used historical healthy controls. Analysis of potential drug effects was limited by insufficient participants not being drug free. Cross-sectional nature of the study limited the analysis of the potential effect of illness variables.

**Conclusions:**

This study replicates earlier findings of increased verbal learning impairment in BDI patients relative to BDII in a substantially larger sample. Such performance cannot be wholly explained by medication effects or illness variables. Thus, the cognitive impairment is likely to reflect a phenotypic difference between bipolar sub-types.

## Highlights

• Largest sample to directly compare cognition in Bipolar I and II subtypes.
• BDI patients impaired relative to BDII on all five verbal memory measures.
• Results remain after adjusting for age, gender, education, mood, and onset age.
• Deficits appear to be unrelated to medication and illness effects.
• Cognitive impairment differences a potential phenotype of bipolar subtypes.

## 1 Introduction

Bipolar disorder (BD) has long been associated with cognitive impairment even in euthymia (Bearden et al., 2001, Martínez-Arán et al., 2004a, Martinez-Aran et al., 2004b, Quraishi and Frangou, 2002, Savitz et al., 2005). Meta-analyses suggest that most domains of cognition exhibit some impairment but that the largest deficits tend to be found in executive control and verbal learning and memory (Bora et al., 2009, Bourne et al., 2013, Goodwin et al., 2008, Kurtz and Gerraty, 2009, Mann-Wrobel et al., 2011, Torres et al., 2007). More recently, studies have attempted to investigate potential differences in cognitive impairment between bipolar I and bipolar II patients (Bora et al., 2011, Hsiao et al., 2009, Simonsen et al., 2008, Solé et al., 2011, Summers et al., 2006, Torrent et al., 2006). Such studies have produced heterogeneous results.

For example, Torrent et al. (2006) compared the performance of 38 euthymic BDI patients, 33 euthymic BDII patients and 35 healthy controls on a variety of neuropsychological assessments. The results showed that both patient groups were impaired on all tests relative to controls but that BDI patients were significantly more impaired than BDII patients on the six outcome measures of the California Verbal Learning Task (CVLT; Delis et al., 1987). In contrast, Summers et al. (2006) found that a group of 11 BDII patients had a verbal memory impairment relative to a group of 25 BDI patients using the Paired Associates Learning Test (PALT; Warrington, 1996), although a proportion of both BD groups were non-euthymic at time of testing. Hsiao et al. (2009) compared the performance of 30 euthymic BDI patients, 37 euthymic BDII patients and 22 healthy controls on a different battery of neuropsychological tests. For some domains, results demonstrated a similar pattern as Torrent et al. but with respect to verbal memory, using the Logical Memory and the Verbal Paired Associates sub-test from Wechsler Memory Scale, third edition (WMS-III; Wechsler, 1997), no impairment was found in BDII patients and the BDI group was impaired relative to both BDII and controls. Similarly, Simonsen et al. (2008) compared 42 euthymic BDI patients, 31 euthymic BDII patients and 124 healthy controls on another neuropsychological test battery and showed that BDII were not impaired relative to controls on both CVLT and Logical Memory tests of verbal memory but that BDI were impaired relative to both BDII and controls.

A systematic review of primary studies of cognition in BDII (Solé et al., 2011) reflected this uncertainty as to whether or not BDII patients exhibit an impairment in verbal memory relative to healthy controls and whether BDII patients are more or less impaired in this domain than BDI patients. Overall, Sole et al. suggest that BDII patients do exhibit cognitive impairment relative to controls but that the severity and pattern of impairment differs from BDI patients. In particular, with respect to verbal learning and memory, the review reports that approximately half the primary studies (four out of nine) found that BDII patients performed worse than healthy controls and that two studies showed BDI patients were more impaired than BDII patients. These findings together suggest that BDII patients may indeed have an intermediate level of impairment relative to healthy controls and BDI patients.

Finally, Bora et al. (2011) conducted a meta-analysis of primary studies of cognitive deficits in BDI and BDII patients and healthy controls. This analysis suggested that both BDI and BDII patients were impaired relative to controls in all cognitive domains considered and that overall there was no difference in performance between BDI and BDII patients in global cognition or in most specific cognitive domains (i.e. processing speed; attention; planning; and working memory). However, a significant difference was found between BDI and BDII patient in verbal memory with BDI patients more severely impaired (E.S.=.48–.53).

All of the studies and reviews that have considered cognitive impairment in BDII patients have been interested in describing a cognitive endophenotype for each subtype that could be the result of underlying neurobiological differences in the subtypes. However, the studies have also noted the potential confounding effects of variables related to illness presentation (such as greater residual depression in euthymic BDII patients) and treatment (greater use of antipsychotics in BDI patients).

The current study investigated cognitive performance of BDI and BDII patients on a verbal learning and memory task in a relatively large patient sample (*N*=279) in an attempt to clarify the existing literature.

## 2 Methods

### 2.1 Study design and sample

Primary data were collected from a cohort of bipolar patients predominantly residing in Oxfordshire and Buckinghamshire in the UK who were participants in the OXTEXT research programme. Demographic and clinical variables were collected for the sample including: (i) age; (ii) years of education; (iii) current mood; (iv) age at onset; (v) number of prior episodes of mood elevation (i.e. manic or hypomanic); (vi) number of prior depressed episodes; (vii) number of prior manic hospitalisations; (viii) number of prior depressed hospitalisations; and (ix) drug treatment history. Participants undertook a very brief battery consisting of four neuropsychological tests, although data presented here are only from an adapted form of the Rey Verbal Learning Task (Rey, 1941) as this cognitive domain appears to show the most consistent inter-subtype differences and also seems to be sensitive to illness and drug effects (Bourne et al., 2013).

The study received ethical approval from a local National Research Ethics Service committee (South Central – Oxford A; REC reference 10/H0604/13) of the UK National Health Service. Each participant was required to provide written confirmation of their consent to participate.

### 2.2 Participants

A total of 279 participants were available for analysis, which comprised 183 BDI and 96 BDII out-patients. All patients had a DSM-IV diagnosis of bipolar disorder. The diagnosis was confirmed by an adaptation of the Mini-International Neuropsychiatric Interview (MINI; Sheehan et al., 1998) and endorsed by a consultant psychiatrist. Although this study did not recruit a healthy control comparison group, a *post-hoc* analysis was undertaken that used healthy controls taken from two previously published cohorts (Cavanagh et al., 2002, Clark et al., 2002). This sample of 50 healthy controls had a mean age of 39.4 years.

### 2.3 Neuropsychological assessment

Five different outcome measures from the verbal learning and memory task are reported: total score on trials 1 to 5 (Total 1–5); score on Short Delay (ShortDelay); score on Long Delay (LongDelay); score on Recognition (Recognition); score for Recognition minus score for False Positives (Recog-FP). Three other cognitive tasks were also used although the data from these other tasks are not reported here. The other tasks in the brief battery of neuropsychological tests were: an emotional face recognition task and an emotional memory task both taken from the Emotional Test Battery (Harmer et al., 2009); and a risk reward judgment task (Abdellaoui et al., 2008). The total duration of the test battery was less than 90 min.

### 2.4 Mood assessment

Participants were invited to enrol in the OXTEXT programme only if their medical notes indicated mood stability over several months. All participants were registered to submit mood ratings on a weekly basis by answering text or email prompts from the True Colours self-monitoring system (www.truecolours.nhs.uk; Miklowitz et al., 2012).

Weekly depression ratings were captured with the Quick Inventory of Depressive Symptomology (QIDS; Rush et al., 2003) and weekly mood elevation ratings with the Altman Self-Rating Mania scale (ASRM; Altman et al., 1997). Mood ratings used in this study relate to the participants rating for the week they undertook the neuropsychological assessment.

Finally, all participants reported were considered to be euthymic by a consultant psychiatrist at time of testing.

### 2.5 Statistical analyses

Parametric statistical tests were used to compare a variety of demographic and illness variables between the two bipolar patients groups. Where appropriate, homogeneity of variance was checked using Levene׳s test.

We investigated any diagnosis effect (BDI v BDII) by regressing diagnosis along with age, gender, years of education, mood (both depression and mood elevation scores) and age of illness onset against performance score for each of the five outcome measures. Years of education was used as a proxy for pre-morbid IQ as these variables have been previously shown to be highly correlated (Bourne et al., 2013). We covaried for variables such as age, gender and education as, whilst these variables may not be significantly different on a between group comparison, they are known to affect neurocognitive performance and can thus reduce the between group effect size (Bourne et al., 2013).

In order to investigate potential drug effects on subtype performance differences, patients were coded for six binary (yes/no) drug status variables: lithium, anticonvulsants, antipsychotics, antidepressants, benzodiazepines and drug free. Each drug status variable together with diagnosis, age, gender, years of education, mood and age of onset were then regressed against performance score for each of the five outcome measures except for drug free as this had less than 10% of patients in one of the drug categories. Drug data was available for between 82% and 85% of our sample depending on the drug type.

Similarly, in order to investigate potential effects of illness severity measures on cognitive performance, number of depressed episodes, number of episodes of mood elevation, total number of episodes, number of hospitalisations due to depression, number of hospitalisations due to mania and total number of hospitalisations were each fitted separately into the regression model with diagnosis, age, gender, years of education, mood and age of onset for each of the five outcome measures.

Statistical analysis was conducted in SPSS version 21 and all statistical tests were two-tailed.

## 3 Results

Table 1a, Table 1b show the demographic and illness profiles of the two diagnosis groups. Overall, the groups were well matched for gender [*χ*^2^(1)=.19, *p*=.66 ], age [*t*(277)=.41, *p*=.68], years of education [*t*(270)=1.36, *p*=.18], mood elevation score [*t*(202)=.71, *p*=.48], and age of onset [*t*(270)=.05, *p*=.96]. However, the groups did differ in depression score [*t*(203)=2.83, *p*=.005] with BDII having a significantly higher level of residual depression. Similarly, the groups differed on the prevalence of antidepressant treatment [*χ*^2^(1)=5.57, *p*=.018] with 32% of BDI patients and 48% of BDII patients on antidepressants. There was a trend for a difference in prevalence of Lithium treatment [*χ*^2^(1)=3.68, *p*=.055] with BDI patients more likely to be taking lithium (37% BDI vs 25% BDII). However, the groups did not differ on prevalence of antipsychotic treatment [*χ*^2^(1)=2.06, *p*=.15], anticonvulsant treatment [*χ*^2^(1)=.20, *p*=.67], benzodiazepine treatment [*χ*^2^(1)=.003, *p*=.96], or drug free status [*χ*^2^(1)=.012, *p*=.91].

The groups did not differ on number of episodes of mood elevation [*t*(238)=1.5, *p*=.13] but did on number of depressive episodes [*t*(241)=2.31, *p*=.02] and total episodes [*t*(235)=2.27, *p*=.03], with BDII patients having more depressive episodes and consequently more total episodes. In contrast, the groups did not differ on number of depressive hospitalisations [*t*(266)=1.3, *p*=.18]. By definition, none of the BDII patients had any manic hospitalisations and because the BDI patients had significantly more manic hospitalisations than zero [*t*(267)=8.05, *p*<.001], total hospitalisations were also more common for BDI [*t*(264)=4.9, *p*<.001].

### 3.1 Diagnosis effects

The results of the regressions comparing performance of the BDI and BDII groups on the five outcome measures whilst also accounting for the effects of age, gender, years of education, mood, and age of onset are shown in Table 2.

The BDII group performed significantly better than the BDI group on all five outcome measures with relatively small but consistent effect sizes (.13–.17).

The covariant age of onset significantly predicted performance on three of the five VLT measures [Total 1–5: *β*=−.19, *t*=2.70, *p*=.008; Long Delay: *β*=−.17, *t*=2.36, *p*=.019; Recognition: *β*=−.22, *t*=2.84, *p*=.005] with early age of onset associated with poorer performance.

### 3.2 Drug effects

The regression analysis comparing the performance of the patients on Lithium and those Lithium free on the five outcome measures whilst also accounting for the effects of diagnosis, age, gender, years of education, mood, and age of onset suggested that lithium did not affect any of the outcome measures (*p*=.25 −.72 for all lithium *β* coefficients). Similarly, antidepressants showed no effect on performance (given effects of diagnosis, age, gender, years of education, mood, and age of onset) on any of the five outcome measures (*p*=.27–.92 for all antidepressant *β* coefficients) and anticonvulsants also showed no effect on performance (given effects of diagnosis, age, gender, years of education, mood, and age of onset) on any of the five outcome measures (*p*=.66−.97 for all anticonvulsant *β* coefficients). Finally, antipsychotics showed no effect on performance (given effects of diagnosis, age, gender, years of education, mood, and age of onset) on any of the five outcome measures (*p*=.18–.55 for all *β* antipsychotic coefficients). Benzodiazepines showed an effect on performance for Total 1–5 scores [*β*=−.14; *t*=2.13, *p*=.035] (given effects of diagnosis, age, gender, years of education, mood, and age of onset) with taking benzodiazepines being related to poorer performance but had no effect on any of the other performance measures (*p*=.26–.33 for all other benzodiazepine *β* coefficients).

### 3.3 Effect of illness variables

The analysis considering the effect of illness variables on VLT performance suggests that most of the variables considered did not affect any of the five outcome measures after accounting for the effects of diagnosis, age, gender, years of education, mood, and age of onset [number of depressive episodes, *p*=.35 −.78 for *β* values across the five VLT measures; total episodes, *p*=.15–.92 for all *β* values; number of depressive hospitalisations, *p*=.11–.92 for all *β* values; number of manic hospitalisations, *p*=.22–.96 for all *β* values; total hospitalisations, *p*=.11–.95 for all *β* values]. Number of episodes of elevated mood was related to performance on Recog-FP [*β*=.21; *t*=2.63, *p*=.009] with more episodes predicting poorer performance as a result of increased false positives. However, number of episodes of elevated mood was not related to performance on any of the other four outcome measures [*p*=.21–.93 for all other *β* values].

## 4 Discussion

The analysis comparing this sample of BDI and BDII patients suggests that BDI patients are more severely impaired on all five VLT outcome measures after accounting for the effects of age, gender, years of education, mood (both depression and mania scores) and age of illness onset. This finding is consistent with the results of several primary studies (Hsiao et al., 2009, Simonsen et al., 2008, Torrent et al., 2006) and a meta-analysis (Bora et al., 2011) with all suggesting that BDII patients outperform BDI patients on verbal learning and memory tests. However, it should be noted that our effect sizes (.13–.17) were considerably lower than those previously found (.48–.53). This was probably due to our partialling-out the effect of age, gender, years of education, mood and age of illness onset on performance.

Indeed, our results are opposite only to that of Summers et al. (2006) where a different verbal memory test was used. Where the literature is less consistent is in comparing the performance of BDII patient with healthy controls; some studies suggest BDII performance is comparable to controls (Hsiao et al., 2009, Torrent et al., 2006) whilst others find BDII patients intermediately impaired relative to both BDI patients and controls (Simonsen et al., 2008, Torrent et al., 2006). As our study had no control group we were unable to directly address this issue. However, we undertook a *post-hoc* comparison of our BDII patients with healthy controls taken from two previous studies of VLT performance in bipolar patients recruited from the same geographical area (Cavanagh et al., 2002, Clark et al., 2002) which used similar duration of test battery. This comparison suggested that our BDII group had an intermediate level of impairment relative to our BDI group and healthy controls. Specifically, our BDII patients were significantly impaired relative to controls after controlling for age and gender on Total1–5 [E.S.=.15; *p*=.045] but performed comparably to controls on the other VLT outcome measures [ShortDelay: E.S.=.09, *p*=.21; LongDelay: E.S.=.10, *p*=.23; Recog.: E.S.=.05, *p*=.55]. These findings are consistent with the mixed picture of prior studies suggesting BDII patients have either an intermediate level of impairment relative to controls or no impairment relative to controls.

Any account of a performance differential between BDI and BDII patients must at this stage be purely speculative. However, two main potential explanations arise. First, BDII is often conceptualised as a ‘milder’ form of BDI: some neurological impairments (relative to controls) appear common to the two sub-types, such as right lateral ventricular enlargements (McDonald et al., 2004) and volume reductions in prefrontal areas (Kempton et al., 2008), especially ventromedial prefrontal areas (Ha et al., 2009), whilst other abnormalities seem present only in BDI patients, such as volume reductions in frontal and parahippocampal cortices (Ha et al., 2009). Indeed, a current mega-analysis (Hibar et al., Submitted) of MRI structural scans from 885 BDI patients, 329 BDII patients and 2613 controls suggests that BDI patients exhibit volume reductions in amygdala, hippocampus, thalamus and ventricular enlargement compared to controls. However, BDII patients showed amygdala, hippocampal and ventricular volumes in between those of controls and BDII patients although not significantly different from either group. It is this intermediate performance of BDII patients relative to BDI patients and controls that we have identified in the verbal learning and memory task. Furthermore, this task involves the hippocampus and left frontal areas associated with language as shown to be impaired in bipolar disorder by neuroimaging studies. Alternatively, intermediate performance of BDII patients may be due to differences in illness progression. By definition, BDII patients do not experience true manic episodes (although our sample shows similar numbers of hypomanic episodes in BDII patients compared to manic episodes in BDI) and cognitive impairment has been shown to be correlated with number of manic episodes (Bourne et al., 2013) especially in verbal learning and memory tasks. Thus, initially both bipolar sub-types may have similar levels of verbal learning and memory performance but that BDI patients suffer further declines in performance, or at least steeper declines relative to BDII, following each manic episode. Finally, it should be acknowledged that these two speculations are not mutually exclusive and may be additive or interactive.

With respect to possible drug effects on performance, our analysis found no basis for the potential claims that lithium may improve cognitive performance via reduced oxidative stress (Mora et al., 2012, Shao et al., 2005, Tan et al., 2011) nor that antipsychotics may reduce performance as some studies have suggested (Donaldson et al., 2003, Jamrozinski et al., 2009). We did find evidence that benzodiazepines might reduce cognitive performance but only for one of the five outcome measures (Total 1–5). Together, these findings are consistent with the recent large IPDMA study (Bourne et al., 2013) that showed that only antipsychotic drugs had any significant effect on cognitive performance and even then only for one of 11 outcome measures considered (albeit within the verbal learning and memory domain).

Although some studies have suggested that illness variables may be predictive of cognitive decline (Robinson and Ferrier, 2006), our findings showed mixed support for this. Age of onset significantly predicted performance for three of the five outcome measures with a lower age of onset being related to worse performance and number of manic episodes being predictive of performance for one outcome measure with more manic episodes being related to worse performance. The finding of a limited effect of age of onset on cognitive performance provides some support for the notion of neuroprogression and clinical staging in bipolar disorder (Berk et al., 2007, Berk et al., 2011). However, for our data to fully support a neuroprogressive model, relationships between illness factors such as illness duration and number of episodes should have been found more widely (Gama et al., 2013). One explanation for a lack of such relationships may be the presence of co-regressors such as age of onset that are highly correlated with key staging/progression variables. Indeed, as noted elsewhere (Bourne et al., 2013), illness progression effects may be difficult to detect, especially in cross-sectional studies and are only likely to result from adequately powered longitudinal studies potentially commencing in pre-clinical, “at-risk” samples. However, the finding of a relationship between manic episodes and more false positives in the recognition task may hint at neuroprogression and related kindling effects (Post, 2007). Clinically, manic episodes are related to increases in impulsivity (Strakowski et al., 2010, Swann et al., 2008) and so individuals exposed to greater numbers of manic episodes may, over time, develop underlying neuronal substrates that promote impulsive behaviours including increased false detection of distractor items in a recognition test (Kockler and Stanford, 2008, Swann et al., 2003).

### 4.1 Limitations

As with any clinical study, a major potential limitation is sampling bias. However, it is noted that the BDI and BDII patient groups presented here were well matched for a variety of confounding variables including age, gender, years of education, mood elevation score, and age of onset. These variables were still used as co-regressors as they are known to affect cognitive performance and we wished to estimate the effect size of sub-diagnosis free from such other effects. Unfortunately, the patient groups were not matched for depression mood score (BDII patients had higher residual mood) or prevalence of lithium medication and antipsychotics medication (BDI patients were more frequently taking both medications). However, depression mood score was also used as a co-regressor in all sub-type analyses and sub-type diagnosis was also included in the drug effect analysis to attempt to control for these confounds. It is also likely that these group differences were confounds by indication and represent underlying differences in symptom presentation and treatment between the two sub-types rather than simple sampling bias. Similarly, the groups were well matched on two further illness variables (number of episodes of mood elevation and number of depressive hospitalisations) but neither on number of depressive episodes and hence number of total episodes (BDII higher for both) nor on the number of manic hospitalisations and hence number of total hospitalisations (BDI higher for both). Again these are considered to be confounds by indication rather than sampling bias per se.

Another limitation of this study is the lack of a specifically recruited healthy control group. However, the *post-hoc* analysis using healthy controls from two prior which used a similar duration of test battery studies and recruited from the same geographical area (Cavanagh et al., 2002, Clark et al., 2002) was intended to mitigate this limitation.

In any event, overall this study finds evidence of significantly greater deficit in BDI relative to BDII patients in all five verbal learning and memory outcome measures. We found no obvious effects of medication and some suggestion that illness variables such as age of onset and number of manic episodes may track cognitive deficits in this domain.

## Role of funding source

This study represents independent research funded by the National Institute for Health Research (NIHR) under its Programme Grants for Applied Research Programme (Reference number RP-PG-0108-10087).

## Conflicts of interests

No conflict declared.

## Authors׳ note

This paper presents independent research funded by the National Institute for Health Research (NIHR) under its Programme Grants for Applied Research Programme (Reference Number RP-PG-0108-10087). The views expressed are those of the author(s) and not necessarily those of the NHS, the NIHR or the Department of Health. Guy Goodwin and John Geddes are NIHR Senior Investigators.

Additional funding for this work was provided by a Wellcome Trust Strategic Award (Collaborative Network for Bipolar Research to Improve Outcomes; Reference Number 102616/Z).

## Figures and Tables

**Table 1a:** Demographic and illness variables for BDI and BDII diagnosis groups.

*N*=279	BD-I (*N*=183)	BD-II (*N*=96)	Comparison test	
	M (SD)	M (SD)	t	p
Age (*N=*279)	41.7 (12.9)	41.0 (14.5)	.42	.71
Years of education (*N=*272)	15.7 (3.5)	15.0 (4.1)	1.36	.18
Mood elevation score *(N=*204)	3.58 (4.4)	4.10 (5.4)	.71	.48
Depression score (*N=*205)	8.79 (5.6)	11.25 (6.1)	**2.83**	**.005**
Age at onset (*N=*272)	20.1 (9.7)	20.0 (10.1)	.05	.96
No. depressive episodes (*N*=205)	19.0 (24.4)	27.7 (30.1)	**2.31**	**.022**
No. manic/hypomanic episodes (*N*=204)	15.5 (21.5)	20.1 (23.8)	1.52	.13
Total episodes (*N*=237)	34.1 (39.4)	48.0 (47.7)	**2.27**	**.025**
No. depressive hospitalisations (*N*=268)	1.4 (2.4)	1.0 (1.9)	1.34	.18
No. manic hospitalisations (*N*=269)	1.5 (2.4)	.0 (.0)	**8.05**	**<.001**
Total hospitalisations (*N*=266)	2.9 (3.6)	1.2 (2.1)	**4.93**	**<.001**

**Table 1b:** Gender and drug status for BDI and BDII diagnosis groups.

*N*=279	BD-I (*N*=183)	BD-II *(N=96)*	Comparison test	
	Count +(−)	Count +(−)	χ^2^	p
Female (Male)	123 (60)	67 (29)	.19	.66
Lithium (*N=*234)	55 (94 free)	21 (64 free)	3.68	.055
Anticonvulsants (*N*=233)	63 (86 free)	33 (51 free)	.20	.66
Antipsychotics (*N*=235)	74 (77 free)	33 (51 free)	2.06	.15
Antidepressants (*N*=231)	47 (100 free)	40 (44 free)	**5.57**	**.018**
Benzodiazepines (*N*=238)	22 (131 free)	12 (73 free)	.01	.91
Drug free (*N*=233)	15 (133 any drug)	9 (76 any drug)	.01	.96

**Table 2:** Comparison of BDI and BDII VLT performance accounting for age, gender, years of education, mood, and age of onset across the five outcome measures.

	BD-I	BD-II			
(*N*=279)	M (SD)	M (SD)	t	p	E.S.
Total 1–5	56.8 (11.2)	58.4 (10.0)	2.16	.032	.14
Short Delay	12.1 (3.0)	12.6 (2.9)	2.01	.046	.13
Long Delay	12.0 (3.3)	12.7 (2.9)	2.05	.042	.14
Recognition	15.0 (1.5)	15.1 (1.4)	2.00	.047	.15
Recog-FP	13.6 (2.9)	14.2 (1.9)	2.36	.020	.17
